# Neuroendocrine Role for VGF

**DOI:** 10.3389/fendo.2015.00003

**Published:** 2015-02-02

**Authors:** Jo E. Lewis, John M. Brameld, Preeti H. Jethwa

**Affiliations:** ^1^Queen’s Medical Centre, School of Life Sciences, University of Nottingham Medical School, Nottingham, UK; ^2^Division of Nutritional Sciences, School of Biosciences, University of Nottingham, Loughborough, UK

**Keywords:** VGF, energy homeostasis, pain, cognition, reproduction

## Abstract

The *vgf* gene (non-acronymic) is highly conserved and was identified on the basis of its rapid induction *in vitro* by nerve growth factor, although can also be induced by brain-derived neurotrophic factor, and glial-derived growth factor. The VGF gene gives rise to a 68 kDa precursor polypeptide, which is induced robustly, relatively selectively and is synthesized exclusively in neuronal and neuroendocrine cells. Post-translational processing by neuroendocrine specific prohormone convertases in these cells results in the production of a number of smaller peptides. The VGF gene and peptides are widely expressed throughout the brain, particularly in the hypothalamus and hippocampus, in peripheral tissues including the pituitary gland, the adrenal glands, and the pancreas, and in the gastrointestinal tract in both the myenteric plexus and in endocrine cells. VGF peptides have been associated with a number of neuroendocrine roles, and in this review, we aim to describe these roles to highlight the importance of VGF as therapeutic target for a number of disorders, particularly those associated with energy metabolism, pain, reproduction, and cognition.

## Introduction

VGF (non-acronymic) is a neurotrophin-induced gene, which was first identified as VGF8a, NGF33.1, and a2 on the basis of its rapid induction in PC12 cells treated with nerve growth factor (NGF) (1–3). Subsequent studies demonstrated that VGF is similarly upregulated by numerous neurotrophins, including brain-derived neurotrophic factor (BDNF) and neurotrophin-3 (NT-3), in neuronal targets such as cortical or hippocampal neurons (4). However, VGF mRNA levels are only marginally increased by other growth factors including epidermal growth factor (EGF), fibroblast growth factor (FGF), interleukin-6 (IL-6), and insulin, despite the capacity of these proteins to robustly induce transcription of other immediate early genes in the PC12 cell line (3, 5, 6).

The VGF polypeptide, which is robustly and exclusively synthesized in neuronal and neuroendocrine cells (1, 3, 7, 8), is processed by the prohormone convertases (PC), PC1/3 and PC2 (9). VGF derived peptides with specific neuronal bioactivities include TLQP-62, TLQP-21, HHPD-41, AQEE-30, AQEE-11, LQEQ-19, and neuroendocrine regulatory peptides-1 and -2 (NERP-1 and -2; 9–11). Studies have shown that TLQP-62 and AQEE-30 increase the firing rate of hippocampal neurons, induce neurogenesis, and have anti-depressive properties (4, 12, 13), whereas HHPD-41, AQEE-30, AQEE-11, and LQEQ-19 stimulate sympathetic outflow and facilitate penile erection in rats (14–16); and TLQP-21 and NERP-2 regulate energy balance (17–20). Furthermore, TLQP-21 regulates contractile activity in the gastrointestinal tract, has analgesic properties, reduces neuronal apoptosis *in vitro* and decreases rodent blood pressure (21–23) and NERP-1 and -2 regulate water homeostasis and suppress vasopressin release (11, 20, 24). Here, we review the regulation of VGF and the neuroendocrine role of its derived peptides.

## The Transcriptional Regulation of VGF

### *In* *vitro*

The gene itself is highly conserved among mammalian species in respect to the coding region and the promoter sequence (25). The VGF promoter region contains a CCAAT box, various specificity protein 1 (SP-1), and activating protein 2 (AP-2) sites and a silencer element similar to the one involved in tissue-specific expression of neuronal genes (3, 25). Furthermore, it contains a cyclic AMP response element (CRE), which is embedded within a 14bp palindromic sequence, mutations of which abolish NGF and cAMP responses (6). VGF expression in response to neurotrophins that requires the combined actions of several regulator complexes; in addition to the CRE, the CCAAT box was shown to be important for NGF induction (26), possibly in association with the activity of a large complex containing a CRE binding protein (CREB), mammalian achaete-scute homolog-1 (MASH-1), and p300 (27).

### *In* *vivo*

A genomic fragment extending from 800-bp 5’ to the transcriptional start site and including the first 700-bp of 5’-untranslated sequence results in reporter gene expression in a tissue-restricted pattern similar to that of the endogenous VGF gene (28). Interestingly, this region of the promoter contains a putative silencer element that is located 400-bp 5’ to the transcriptional start site, which prevents expression in non-neuronal cell lines (25). VGF mRNA in the hypothalamus alters in response to feeding/fasting (14, 15, 20), salt loading (29), adrenalectomy (30), and seasonal rhythms (31). Furthermore, VGF mRNA varies in the pituitary during the estrous cycle (32) and in the suprachiasmatic nucleus (SCN) according to circadian rhythmicity (33); while gastric damage increases VGF mRNA in the nucleus tractus solitarius (NTS) and dorsomedial nucleus of the vagus (34). VGF mRNA is also modulated in other diverse conditions, which have been well described elsewhere (35).

## The Structure and Processing of the VGF Polypeptide

**Figure 1:**
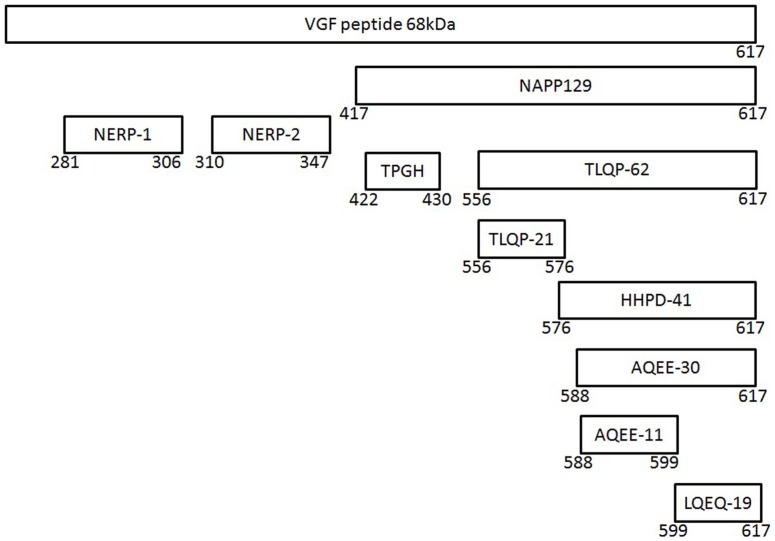
**The *vgf* gene and its derived peptides**. The VGF polypeptide is the precursor of several biologically active peptides, which are released and play a role in intercellular communication. The gene contains a number of specific sequences, which are highly conserved between the species and these represent potential cleavage sites for the convertases of the kexin/subtilisin-like series proteinases family, namely prohormone convertases-1/3 and -2.

**Figure 2:**
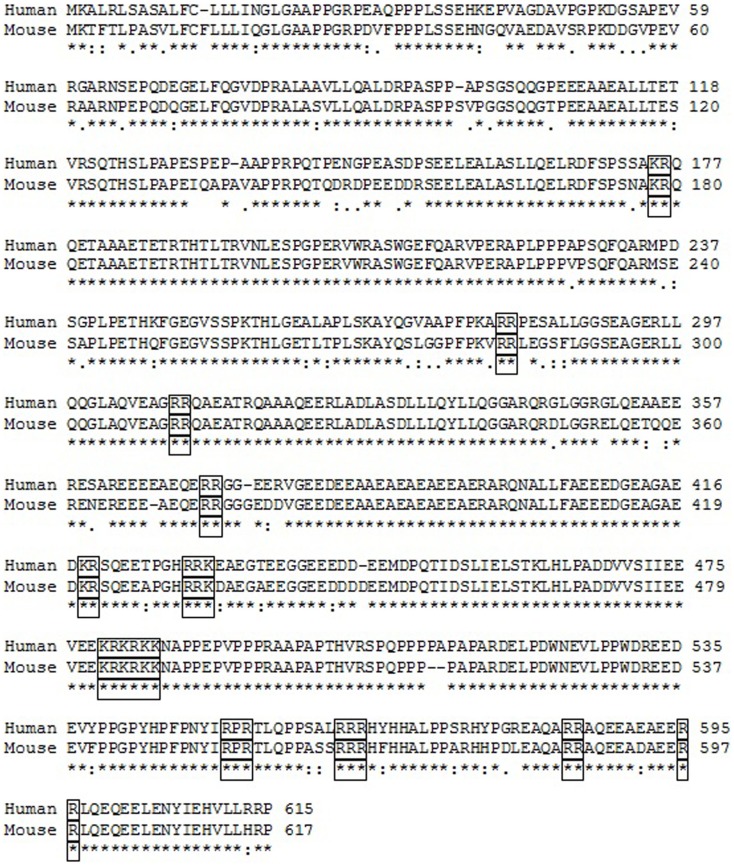
**Comparison of the human and mouse VGF polypeptide sequences**. * indicates the position which have a single, fully conserved residue. “:” indicates conservation between groups of strongly similar properties scoring >0.5 in the Gonnect PAM 250 matrix. “ . ” indicates conservation between groups of weakly similar properties scoring <0.5 in the Gonnect PAM 250 matrix. Clusters of basic amino acids, which represent potential cleavage sites, are boxed. Sequence identity was >85%.

VGF is a 68 kDa polypeptide comprising 615 (human) or 617 (mouse/rat) amino acids with a typical secretory leader sequence of 22 amino acids at the N-terminal of VGF, which promotes translocation to the endoplasmic reticulum (ER) (36). Subsequent sequencing of the polypeptide in the mouse, horse, and bovine has confirmed extensive sequence conservation with approximately >85% identity (35). The most prominent VGF-derived peptides have apparent molecular masses of 20 (NAPP-129) and 10 kDa (TLQP-62), respectively (9) (Figure 1). However, the mouse and human sequences contain a minimum of 10 conserved regions of basic amino acid residues, which represent potential PC cleavage sites (37) (Figure 2). Indeed cleavage at the Arg-Pro-Arg_555_ sequence in the rat has been shown to give rise to the TLQP peptides (9). It is possible, however, that the number and function of VGF derived peptides are greater than currently known (38). The extensive review by Ferri et al. (35) describes this in more detail.

## Distribution of VGF and Its Derived Peptides

### VGF mRNA

VGF mRNA is widely expressed throughout the nervous system. During embryogenesis VGF mRNA is expressed in distinct neurotrophin-responsive targets in the central and peripheral nervous system (CNS and PNS, respectively) in the rat (39, 40). At birth, VGF mRNA is expressed in neurons throughout the brain and in peripheral endocrine and neuroendocrine tissues. While in the adult brain VGF mRNA has the highest expression in the hypothalamus and the granular layer of the cerebellum, it is also expressed in a number of other brain areas including the main and accessory olfactory bulbs, hippocampus, cortex, basal ganglia, thalamus, amygdala, midbrain, and the brainstem. Within the hypothalamus, the highest concentrations of VGF mRNA have been found in the ventromedial hypothalamus, in particular the arcuate nucleus (ARC), as well as in the SCN (7, 39, 41). VGF mRNA expression in the mouse is similar to the rat (14).

### VGF peptides

VGF and its derived peptides are found in dense core vesicles and are released in response to depolarizing signals from neuronal and neuroendocrine cells through the regulated secretory pathway (10, 42, 43). Antibodies raised to synthetic peptides corresponding to the C- or N-termini of potential or actual cleavage products have been utilized to study VGF derived peptide distribution. In animal tissues, VGF immunoreactivity was restricted to central and peripheral neurons (41, 44), as well as to endocrine cells of the pituitary, adrenal medulla, gut, and pancreas (44). The highest concentrations of VGF immunoreactivity have correspondingly been found in the medial hypothalamus, particularly in the ARC, in the SCN, and in the parvocellular and magnocellular cells of the paraventricular nucleus (PVN) and the supraoptic nucleus (SON) (41). Weak immunoreactivity was also detected in the hippocampus, amygdala, thalamus, and cerebral cortex (41). VGF immunoreactivity was also displayed in the female rat in the pars distalis, mainly with C-terminal antibodies (32). This, however, disappeared in accordance with the estrous peak of luteinizing hormone (LH) secretion, along with an induction of VGF mRNA in the pituitary. Additionally, VGF-derived peptides are prominent in the adult spinal cord, in α- and γ-motor neurons of the ventral horn and in the dorsal horn neurons, as well as cells of the inner nuclear and ganglion cell layers of the retina (45). In the PNS, both sympathetic ganglia and dorsal root ganglia of primary sensory neurons are important sites of localization of VGF-derived peptides (40). This is comparable to the expression of neuropeptide Y (NPY), ghrelin, and cholecystokinin (CCK), all of which regulate feeding and in some cases, gastrointestinal motility (46, 47). VGF-derived peptides are also present in mouse brown adipose tissue (BAT), where they are reduced in response to a high fat diet (HFD) (48).

## VGF Receptors

Of all the VGF derived peptides, TLQP-21 has had the most interest (17, 19, 23, 49–52). Previously, TLQP-21 was shown to bind to adipocyte membranes in a saturable manner, (53) and atomic force microscopy of living cells revealed the existence of a single class of binding sites for TLQP-21 (54). Taken together these results suggested a cell surface receptor for TLQP-21. Two possible receptors have recently been identified for TLQP-21. Chen et al. (55) identified gC1qR, showing that TLQP-21 activated rat macrophages through gC1qR, which then caused mechanical hypersensitivity in rats. gC1qR protein was expressed by both brain and spinal cord derived microglia (55) and is indispensable for adipogenesis and insulin signaling (56). Furthermore, obese mice fed a HFD demonstrated increased density of TLQP-21 binding in adipose tissues (54). However, neither TLQP-62 nor LQEQ-19 elicited a response in their experimental model, both of which had been previously implicated in pain processing (22, 57). This supports the hypothesis of different receptors for the VGF derived peptides. Hannedouche et al. (58) reported the complement receptor, C3A receptor-1 (C3AR1), as a receptor for TLQP-21, which mediated activity for TLQP-21 in two different rodent cell lines. C3AR1 was originally thought to be restricted to the innate immune response, its role limited to the complement cascade. However, it has subsequently been shown to have a role in cancer (59), neurogenesis (60), and hormone release from the pituitary gland (61). However, C3AR1^-/-^ mice are transiently resistant to diet-induced obesity (DIO) and are protected against HFD-induced insulin resistance (62). The discovery of these receptors will help identify the mechanisms by which TLQP-21 and possible other derived peptide may modulate its actions.

## Physiological Roles of VGF Gene and Derived Peptides

### Energy balance

The high expression of VGF in the hypothalamus and the change in expression of the *vgf* gene in the ARC following acute altered energy balance first suggested the importance of VGF in the regulation of energy balance (14, 20). Indeed fasting has been shown to increase VGF mRNA expression, while administration of leptin prevents the fasting induced increase in VGF mRNA (15). These changes in VGF can be observed in models of chronic energy imbalance; VGF mRNA levels resemble that of fasted wild-type mice in the ARC of the leptin deficient *ob/ob* mouse and in the leptin resistance *db/db* mouse (15). It is well known that the ARC has two neuronal populations that respond to the fed and fasted state as well as to leptin signaling, the pro-opiomelanocortin (POMC) and neuropeptide Y (NPY) neurons (63). VGF immunoreactivity has been shown to be co-localized with both these neuronal populations in the ARC, however, expression is modulated with energy state. In the *ad libitum* fed state and re-fed animals, VGF mRNA is co-localized with POMC (15, 64). On the other hand, fasting increases co-localization of VGF in the NPY neurons (64).

#### Energy balance and lack of functional VGF

The function of VGF and its extension derived peptides was first assessed through the development of mice lacking a functional copy of the *vgf* gene (VGF^-/-^) via homologous recombination (14). At birth, the homozygous VGF^-/-^ mice are indistinguishable from either their heterozygous or wild-type littermates. No defects in development were detected in either the CNS or the PNS. However, in the weeks following birth, the VGF^-/-^ mice were visibly smaller than their wild-type littermates and adults were found to weigh 50-70% less due to a 50% reduction in adiposity compared to wild-type littermates (14). Consistent with the reduction in adiposity, leptin levels, serum glucose and insulin levels, and liver glycogen were reduced (48). The mice consumed considerably more calories per gram body weight, but this increase in food intake was not sufficient to maintain the same body weight as wild-type mice. The VGF^-/-^ mice utilized twice as much oxygen at rest and displayed increased locomotor activity compared to wild-type littermates (14). Overall, the major change in VGF^-/-^ mice is an increase in energy consumption; indeed *vgf* gene deletion did not block obesity via monosodium glutamate administration (15) suggesting that the thermogenic pathways resulting in the VGF^-/-^ phenotype are blocked. These initial observations led Hahm et al. (14) to suggest that VGF may play a non-redundant role in the regulation of energy homeostasis and antagonism of the gene may constitute a basis for the treatment of obesity. Furthermore, *vgf* gene deletion blocked the development of obesity as a result of a HFD, gold thioglucose treatment, as well as in the *agouti* mouse, and suggesting that VGF functions in outflow pathways regulating energy expenditure downstream of the hypothalamic melanocortin receptors (15).

#### Energy balance and VGF-derived peptides

Thus from the phenotype of the VGF^-/-^ mice one might predict that VGF promotes an anabolic drive. Surprisingly, this view has not been supported by subsequent studies in mice and Siberian hamsters. Chronic intracerebroventricular (ICV) infusion of TLQP-21 in mice fed a normal lab chow resulted in a small increase in resting energy expenditure and rectal temperature (17). The changes in metabolic parameters were mirrored by increased epinephrine content in BAT, upregulation of BAT β2-adrenergic receptor (AR), uncoupling protein 1 (UCP-1) mRNA, higher expression of peroxisome proliferator-activated receptor-δ (PPAR-δ), and β3-AR in white adipose tissue (WAT). However, hypothalamic expressions of agouti-related protein (AgRP), NPY, α-melanocyte-stimulating hormone (α-MSH), POMC, and corticotrophin-releasing hormone (CRH) were unchanged (17). In mice, switched to a HFD treatment with TLQP-21 halted the expected increase in body weight and WAT, attenuated rises in leptin, and normalized ghrelin levels (17). In rats, ICV infusion of TLQP-21 significantly decreased gastric emptying, an effect that was blocked by ICV infusion of indomethacin, which blocks prostaglandin release (65).

A similar catabolic effect was noted in Siberian hamsters, a seasonal model of energy balance. Not only is VGF mRNA significantly increased in the winter weight-loss state in the dorsal medial posterior arcuate nucleus (dmpArc) (31) but ICV infusion of TLQP-21 at the onset of the dark phase was found to significantly and dose dependently decrease food intake and body weight (19). However, there was no effect on energy expenditure as Siberian hamsters pair-fed to the treated group lost a similar amount of body weight (19). Weight loss was, therefore, attributable to reduced caloric intake rather than energy expenditure.

One of the possible explanations for this contradiction between the functional *in vivo* studies and the VGF^-/-^ mice, where all the VGF peptides have been ablated, is that some of these peptides may have opposing roles in energy balance. Interestingly, Bartolomucci et al. (66) have suggested that HHPD-41 increased food intake following ICV infusion, and more recently ICV infusion of NERP-2 in rats has been shown to increase food intake, body temperature, oxygen consumption, and locomotor activity (20). Furthermore, intravenous administration of NERP-2 significantly augmented glucose stimulated insulin secretion in anesthetized rats or following intraperitoneal injection to conscious mice (67). Thus VGF may have a biphasic role in the regulation of energy balance and further characterization of the other VGF-derived peptides is required.

#### Energy balance and circadian rhythm

It is well known that food intake and energy metabolism in mammals are regulated by their circadian clock, and food intake is one such signal that can entrain the circadian clock (68). As previously described, VGF is expressed in the SCN, the circadian pacemaker in animals, while the E-box contained in the *vgf* gene promoter region is similar to the many clock genes such as the *per* gene (33). Therefore, it is not unexpected that the *vgf* gene exhibits circadian rhythm in the SCN even under constant dark conditions, while VGF mRNA levels are increased in response to light simulation in the SCN when light would be expected to cause a phase shift in locomotor rhythms (33). Indeed VGF^-/-^ mice can maintain circadian rhythm of wheel running in constant darkness, however, the period length was found to be slightly but significantly shorter than wild-type littermates (14). Thus, this raises the question could the metabolic phenotype of the VGF^-/-^ mice be attributed, in part, to the disruption of the circadian system.

### VGF and water balance

Water deprivation and salt loading in rats increases VGF mRNA levels in both the SON and PVN, along with vasopressin mRNA (29). ICV injection of NERP-1 and NERP-2 suppresses hypertonic saline or angiotensin II induced increases in plasma vasopressin in rats (69). Additionally, ICV infusion of NERP-1 and -2 attenuated the increase in vasopressin as a result of water deprivation in rats, an effect which was reversed following immunoneutralisation by ICV infusion of anti-NERP-1 and -2 antibodies (69). Taken together, these data suggest that NERP-1 and -2 may be involved in the central control of body fluid balance.

### VGF and reproduction

The role of VGF signaling in reproduction was inferred from the observation that VGF gene deletion resulted in infertility in both male and female mice (14). In male VGF^-/-^ mice, the onset of puberty and sexual maturation was delayed, and the weights of the testes, albeit having mobile spermatozoa in the lumen, were significantly lower than those of wild-type littermates (14). While in the female VGF^-/-^ mice histological examination revealed no mature follicles or corpus lutea, and the ovaries, ovidut, and uteri weighed 30% less than those of the wild-type littermates (14). However, transplanting ovaries from VGF^-/-^ mice into ovariectomized wild-type females restored fertility, suggesting that the reproductive deficits of VGF^-/-^ mice were not the result of pathology but arose from deficits in the hypothalamic-pituitary-gonadal axis (14). However, Ferri et al. (32) showed that VGF gene expression varied during estrous; there was an increase in VGF mRNA and VGF peptide/s degranulation, suggesting perturbation of anterior pituitary function.

It is common knowledge that alterations in energy metabolism and fat stores can affect reproductive function. VGF^-/-^ mice have reduced leptin and altered energy status, therefore, it could be suggested that the deficit may be due to gonadotropin releasing hormone (GnRH) synthesis or secretion. However, while GnRH levels are not affected, LH and follicle-stimulating hormone (FSH) mRNA levels were reduced in VGF^-/-^ mice (14) suggesting decreased GnRH secretion. Indeed it has been shown that central administration of TLQP-21 in female rats during the pubertal transition advanced the timing of vaginal opening and increased the number of animals with signs of ovulation (70). These effects of TLQP-21 may be via stimulation of the GnRH release, as TLQP-21 has been shown to induce LH secretion *in vitro* (71). Furthermore, Pinilla et al. (71) have shown that chronic administration of TLQP-21 was able to prevent the hypogonadotropic state induced by food deprivation.

There is further evidence of VGF peptides and a possible role in the regulation of reproduction. While HHPD-41, AQEE-30, and LQEQ-19 have been shown to induce penile erection in rats following infusion into the PVN in a dose dependent manner, NERP-1 has a pro-erectile effect when injected into the lateral ventricles or the ARC of rats (72). The effect on penile erection is thought to be via nitric oxide mediated activation of oxytocinergic pathways (16).

### VGF and pain

VGF is a gene commonly upregulated in sensory neurons in clinically relevant models of neuropathic pain, namely, varicella zoster infection, HIV-associated neuropathy, and peripheral nerve trauma (55). Furthermore, VGF has been shown to be upregulated in the dorsal root ganglia and spinal cord in a number of neuropathic and inflammatory pain models (22, 57, 73–76). In these areas, VGF is co-localized with substance P, calcitonin gene related peptide, TrkA, and P2 × 3 (22, 51). A functional role for VGF-derived peptides has been identified in pain pathways. Indeed intrathecal infusion of TLQP-62 results in cold behavioral hypersensitivity in rats; while injection of TLQP-21 into the hind paw of mice resulted in hypersensitivity in both control animals and the formalin model of inflammatory pain (51) as well as inducing thermal hyperalgesia in the warm-water immersion tail-withdrawal test (77). Additionally, both LQEQ-19 and AQEE-30 have been shown to induce p38 MAP kinase phosphorylation in spinal microglia (22), suggesting that VGF-derived peptides have pro-nociceptive and hyperalgesic functions.

### VGF and memory and learning

As previously stated VGF mRNA is expressed in the hippocampus, and it has been shown that VGF transcription is accompanied by translation within 3 hours of BDNF exposure in hippocampal slices *in vitro* (4). Additionally, VGF mRNA has been shown to be upregulated by activities, such as memory and learning (8), while VGF^-/-^ mice have demonstrated impaired hippocampal-dependent spatial learning and contextual fear conditioning tasks (78). Indeed more recently, TLQP-62 has been shown to induce transient potentiation in hippocampal slices (78), enhance synaptic activity (4), and increase neurogenesis in early phase neural progenitor cells in the adult hippocampus (12, 79), as well as shown to have effect on cognitive mechanism (80), thus suggesting that VGF may be important in memory processes.

To further support this notion, proteomic studies have demonstrated a reduction in VGF-derived peptides in the cerebrospinal fluid of patients affected by Alzheimer’s disease (AD) (81–83). Similarly, there was a reduction in VGF-derived peptides in the parietal cortex of AD patients (84) and a reduction in TPGH and NERP-1 in the parietal cortex of Parkinson’s disease patients (84).

### VGF and depression

VGF protein expression is reduced in both the learned helplessness and forced swim test depression paradigms (85), while VGF is increased by antidepressant drugs and voluntary exercise (12). Exercise regulates VGF mRNA and protein expression in the rodent hippocampus and induces an antidepressant response; an opposing phenotype is observed in the heterozygous VGF^-/+^ mouse (86). Recently, inhibition of phosphodiesterase-4 or -5 was shown to result in increases in cAMP, activating CREB, BDNF, and VGF, which produces antidepressant-like effects on behavior in mice (87). Similarly, microinjection of TLQP-62 into the hippocampal CA1 regions demonstrated antidepressant-like behavioral effects in mice (88), possibly via a BDNF-dependent mechanism (78).

## Conclusion

The evidence presented in this review indicates that the gene and gene product have a key neuroendocrine role and that VGF or its derived peptides may act as biomarkers or therapeutic targets in a number of disorders such as obesity, dementia, depression, and pain. The mechanisms by which VGF and its derived peptides are involved remains to be identified, however, the discovery of the new receptors will help advancements in this area both *in vitro* and *in vivo*.

## Conflict of Interest Statement

The authors declare that the research was conducted in the absence of any commercial or financial relationships that could be construed as a potential conflict of interest.
